# Fabrication of Polyhedral Particles from Spherical Colloids and Their Self-Assembly into Rotator Phases**

**DOI:** 10.1002/anie.201409594

**Published:** 2014-11-03

**Authors:** Hanumantha Rao Vutukuri, Arnout Imhof, Alfons van Blaaderen

**Affiliations:** Soft Condensed Matter, Debye Institute for Nanomaterials ScienceUtrecht University, Princetonplein 1, 3584 CC, Utrecht (The Netherlands)

**Keywords:** colloids, polyhedral particles, polymers, rotator phases, self-assembly

## Abstract

Particle shape is a critical parameter that plays an important role in self-assembly, for example, in designing targeted complex structures with desired properties. Over the last decades, an unprecedented range of monodisperse nanoparticle systems with control over the shape of the particles have become available. In contrast, the choice of micrometer-sized colloidal building blocks of particles with flat facets, that is, particles with polygonal shapes, is significantly more limited. This can be attributed to the fact that in contrast to nanoparticles, the larger colloids are significantly harder to synthesize as single crystals. It is now shown that a very simple building block, such as a micrometer-sized polymeric spherical colloidal particle, is already enough to fabricate particles with regularly placed flat facets, including completely polygonal shapes with sharp edges. As an illustration that the yields are high enough for further self-assembly studies, the formation of three-dimensional rotator phases of fluorescently labelled, micrometer-sized, and charged rhombic dodecahedron particles was demonstrated. This method for fabricating polyhedral particles opens a new avenue for designing new materials.

Building blocks consisting of non-spherical and anisotropic particles offer a vast variety of structures with different symmetries, packing densities, and directionalities as compared to structures that are built from isotropic spherical particles.[1] In the field of nanoparticles, the amount of new particle model systems with a well-defined complex shape has increased exponentially.[1a,c] The methods reported include photochemical, thermal, electrochemical, and template-directed procedures but almost exclusively deal with nanocrystalline particles.[1a,c] As it is now often possible to predict what structure is needed for certain material properties, the main challenge is to experimentally realize those particle interactions and to develop procedures that lead to the desired structures. The self-assembly of complex shapes with complex interactions provides one possible route to realize the desired structures. The growing demand for micrometer-sized colloidal particles with complex shapes is largely driven by their applications in photonics as well as in advanced functional materials.[1f,g] Several synthetic routes have been reported to fabricate particles with non-spherical geometries. These approaches make use of self-assembly,[2] photolithography,[3] microfluidics,[4] non-wetting template molding,[5] stretching polymer-embedded particle films,[6] thermal sintering of spherical particles at an oil–water interface,[7] template methods,[1g,^8^] metal–organic frameworks,[9] and seeded emulsion polymerization.[10] Collectively, these methods have produced particles of several distinct shapes, including metal-based polyhedral particles. Moreover, flat facets on the particle surface play an important role in self-assembly. For instance, particles tend to align along the flat interfaces at higher densities,[11] especially for hard particle interactions. With a depletant added, this particle geometry enhances directional attractive interactions between two flat interfaces.[3,12] Recent experimental work also indicated that similar arguments hold for van der Waals, electric double-layer, and electric-field-induced interactions.[13] Several recent computational and theoretical studies have predicted that polyhedral colloidal particles show a rich phase behavior, including quasicrystals and rotator phases that still allow full particle rotation in a 3D ordered lattice of the polygons,[1e,h] but experimental realizations of those phases with micrometer-sized colloidal particles scarcely exist. We herein describe the method that we developed for studying these systems quantitatively on the single-particle level.

Over the past few years, we have developed a thermal sintering method for creating permanently bonded 1D colloidal analogues of polymer bead chains,[2a,b] 2D sheets,[14] and 3D structures[15] with sterically stabilized and “unlocked” particles (i.e., the stabilizer molecules are not covalently linked to the particles) of poly(methyl methacrylate) (PMMA).[16] For the particle deformation process presented here to yield individual particles, it was found that the steric comb-graft-type stabilizer molecules must be covalently linked to the polymer chains in the core of the particles (using a so-called locking step).[16] Sintering phenomena, which lead to particle deformation, are known to occur in the intermediate stages of latex film formation,[17] which is used in several industrial processes associated with paints, paper coatings, textiles, and carpets, for example. The deformation of the particles takes place once they are in close contact with their neighbors either in the dry state (dry sintering) and/or in the wet state (wet sintering).[17] Generally, in those stages also some exchange of material from one particle to its neighbors by polymer chain diffusion occurs, but this process mostly takes place in the final stages of film formation. This effectively prohibits the detachment of the deformed particles and the preservation of any smooth surfaces that have formed.[17,18] Herein, we demonstrate that under certain conditions, the sintering process can be carried out without any exchange of material among adjacent particles using sterically stabilized and locked particles,[16a while concurrently effecting the deformation of particles by contact with their direct neighbors. Our method consists of three simple steps (Figure 1): 1) the self-assembly of sterically stabilized and locked polymeric spherical particles into a 3D crystalline structure, 2) the deformation of the spherical particles by thermal annealing in such a way that mass transport between the particles does not take place, and 3) the redispersion of the resulting deformed particles from the 3D superstructures into individual particles by sonication. Moreover, we demonstrate the self-assembly of rhombic dodecahedron particles into rotator phases at different salt concentrations.

**Figure 1:**
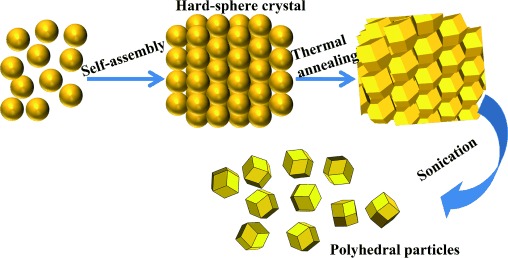
Procedure for the fabrication of polyhedral particles.

Several methods are available for growing colloidal crystals. We chose a simple method, sedimentation-induced crystallization, for our nearly refractive-index-matched, hard-sphere-like[15,19] colloidal suspension of rhodamine isothiocyanate (RITC) labelled PMMA particles in cyclohexyl bromide (CHB). The suspension (particle content: 10–20 % by volume) was transferred to a capillary cell with a 0.2 mm×2.0 mm cross section and of a suitable length (ca. 10 cm) oriented in an upright position. After one to two weeks, a large random hexagonal closed-packed (rhcp) crystal was observed. Although face-centered cubic (fcc) is the thermodynamically most stable phase, the free energy difference with respect to the metastable hexagonal closed-packed (hcp) arrangement is very small (ca. 10^−4^ *k*_B_*T* per particle) at the melting transition. Therefore, a random hcp structure has been observed experimentally more often than pure fcc. However, it is known that pure fcc crystals can be achieved using particles that interact through a slightly soft potential or by using sedimentation on a template if necessary.[20]

Next, the crystals were heat-treated by immersing the capillary in a hot water bath at 75 °C, which is well below the glass transition temperature (*T*_g_=140–145 °C) of bulk PMMA.[15] In wet sintering, the interfacial tension between the particles and the solvent is the dominant driving force. Moreover, this liquid modifies not only the surface tension, but also the glass transition temperature of the PMMA as it induces a slight swelling of the particles.[15] Furthermore, it is known that even for homogeneous polymer particles, the glass transition temperature for the outer layer of the polymer can differ significantly (by tens of degrees) from that of the interior.[18a As a consequence, the touching spheres underwent a plastic deformation in the presence of the solvent. This sintering process has been discussed in the literature using a range of models as arising either from a viscous-flow process driven by surface tension effects or from an elastic Hertzian deformation of elastic spheres under tension.[17,18] Almost certainly the reality is more complex and is likely of a visco-elastic intermediate nature where the details depend mostly on particle and stabilizer properties.[17,18]

During the initial stages of sintering, approximately two to three minutes after the heating was started, the particles were found to form flat facets at the contact points owing to the surface forces between the particles as mediated by the solvent. Samples were subsequently cooled down to room temperature and dried after opening of the capillary for approximately two to three days at room temperature. It can be clearly seen that the crystal building blocks then consisted of non-spherical particles with small patches (Figure 2 a). We exploited the time dependence of the thermal sintering process for creating different particle shapes, mostly spherical particles with small, flat patches (Figure 2 a) at one extreme and polygonal particles (Figure 2 c) with a rhombic dodecahedron shape at the other extreme. Ultimately, the deformation will continue until the particles reach the Voronoi polygons of their colloidal crystal structure, which dictates the symmetries of their deformation. The tunability of the particle shape is exemplified for different sintering times (2–3, 5, and 10 min) in Figure 2 a–c, respectively. For a sintering time of five minutes, the particles already started to deform and flatten where the particles were physically in contact with neighboring particles. As a result, after the heat treatment, each particle had 12 flat patches on its surface. This can be attributed to the fact that each particle has twelve neighbors, six of which are in the same layer and three each in the layers above and below (Figure 2 b). As can be seen in Figure 2 c, a sintering time of ten minutes was already sufficient for the particles to reach their polygonal Voronoi shape and to transform into rhombic dodecahedra. To quantify the flatness of the patches and the facets in more detail, we characterized the particle morphology using atomic force microscopy (AFM; see the Supporting Information, Figure S1) in tapping mode. These measurements confirmed that the patches and the facets were flat and had a root-mean-square roughness of about 9 nm and 4 nm, respectively (see the Supporting Information). Thus, the particle shape only depends on the number and the arrangement of neighboring particles that are in direct contact with the particle and on the heating time. For example, we observed that the particles that were in contact with the wall obtained a different shape (see Figure S2). If the particles are ordered in a fcc array and uniformly deformed for long enough, the resulting particles will be regular rhombic dodecahedra (see Figure 2 c, inset). Otherwise, spheres with a similar symmetric distribution of flat patches will result where the patch diameter can be tuned by the sintering time. Similarly, particles with a simple cubic symmetry are predicted to transform into cubes, body-centered cubic packed particles will turn into truncated octahedra, and hcp particles will transform into hexagonal prisms. As a proof of concept, we sintered a body-centered tetragonal (bct) crystal,[15,19] which was formed by induced dipolar interactions in the presence of an external electric field (see the Supporting Information). The resulting particle shape is shown in Figure 2 d.

**Figure 2:**
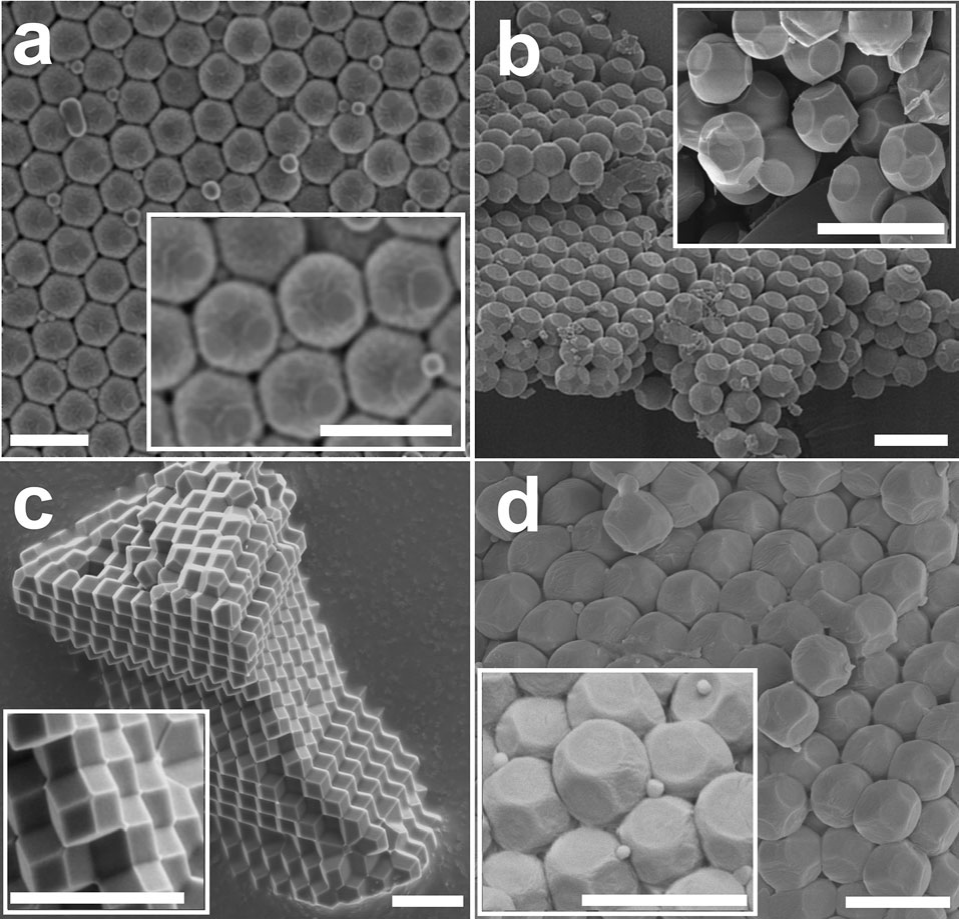
SEM images of dried polyhedral PMMA particles. a–c) Heat-treated and dried hard-sphere fcc crystals of PMMA particles that were annealed at 75 °C for different heating times (*t*_h_) in the presence of the solvent: *t*_h_=2–3 min (a), *t*_h_=5–6 min (b; insets show magnified images of the patchy particles), and *t*_h_=10–12 min (c; inset: magnified image of the rhombic dodecahedron shape). d) Heat-treated bct crystal. SEM clearly reveals the characteristic bct stacking: A square arrangement of spheres is obtained perpendicular to the applied electric field (inset: magnified image of the particles). Scale bars: 5 μm.

Although for the crystals shown in Figure 2 c, d, the particles had completely deformed by the time the packing fraction nearly reached unity, filling all interstitial space in the crystal, it was still possible to resuspend the particles as individual entities by sonicating the annealed crystals for about 10–15 minutes at room temperature in CHB. Apparently, the stabilizer forms an effective protective layer. As the backbone of the stabilizer is a long PMMA chain, its covalent bonding to the particle likely involves bonding with several PMMA molecules in the outer layer of the particle. This would effectively make the stabilizer a cross-linked layer. Additionally, this cross-linked layer completely prevented the inter-diffusion of PMMA polymer chains between the neighboring particles during the thermal annealing, and thus the resulting surfaces of the particles remained remarkably smooth (Figure 2 c, inset). Indeed, sterically stabilized but unlocked particles were unable to separate into the individual entities (see Figure S3).

Clearly, for individual particles, the equilibrium shape is a spherical shape. Fortunately, even after two to three weeks of being dispersed in CHB at room temperature, the particles still retained their polygonal shapes with sharp edges, which became apparent after drying the particles and then examining them by scanning electron microscopy (SEM, see Figure S4). We characterized the morphology of the polyhedral particles in terms of the distribution of their edge lengths (*a*) and the percentage of particles with the intended shape present in the sample from the SEM images using iTEM software (Olympus Soft Imaging Solutions). The edge length of the particles was measured to be 0.676±0.06 μm with a polydispersity of 8.9 %, and 84 % of the particles had a rhombic dodecahedron shape. The yield of the rhombic-dodecahedron-shaped particles is less than 100 % mostly because of stacking errors and other defects and can be drastically improved if the purity of the fcc phase is increased, for instance by making the colloidal crystals using a template (colloidal epitaxy).[20]

Because of the stability of our deformed particles in CHB, these systems can therefore be both index- and density-matched, so that bulk measurements of self-assembly in real space are possible. Several recent simulation studies reported on the mesophase behavior of space-filling hard polyhedrons, namely truncated octahedrons, rhombic dodecahedrons, hexagonal prisms, cubes, gyrobifastigiums, and triangular prisms.[1e,h] For these particle systems, the formation of various new liquid–crystalline and 3D rotator or plastic crystalline phases has been predicted at intermediate volume fractions in simulations.[1e,h] Herein, we show the phase behavior of our sterically stabilized and charged rhombic dodecahedron particles at different salt concentrations to modify the Debye screening length (*κ*^−1^, a measure for the range of repulsion) as compared to the particle size. When the potential between the particles is made soft (*κR*≈1, long-range screened electrostatic repulsions) by dispersing them in a deionized solvent (CHB), crystallization occurs at a small volume fraction (*ϕ*). Figure 3 a clearly illustrates the softness and the hexagonal positional order of the crystalline particles in the plane (111). Owing to the finite resolution (ca. 250 nm in the imaging plane) of the confocal microscope, it is hard to visualize the detailed features of the particle shape. However, it is still clearly visible in Figure 3 a that the particles are indeed non-spherical. Owing to the long-range repulsive interactions between the particles, they undergo free 3D rotations on their lattice positions (see Movie S1). Therefore, the time-averaged image of this rotator phase over a period of 180 s (Figure 3 b) shows the polygons as spherical objects. Moreover, the experimental *g*(*r*) value is in agreement with a 2D hexagonal crystal layer as shown in Figure 3 c. In Figure 3 d, the vertical *xz* slice reveals the ABC stacking sequence, confirming the fcc symmetry of the crystals. The rotator phase is the phase where the particles are randomly oriented with respect to each other (no long-range orientational order), but the centers of mass of the particles are ordered in a crystalline structure (long-range positional order).[21]

**Figure 3:**
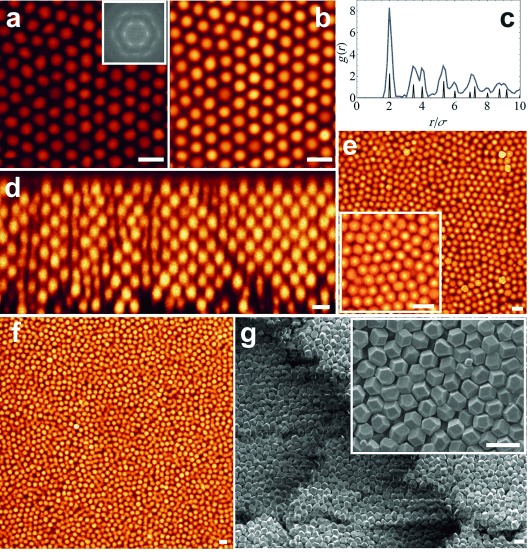
Plastic crystals, or rotator phases, of rhombic dodecahedron PMMA particles. a) Confocal *xy* snapshot of the fcc (111) plane of a rotator phase of PMMA particles in deionized CHB. Inset: Fourier transform calculated from the real-space image (*xy*). b) Confocal *xy* image time-averaged over 180 s. c) In-plane (2D) radial distribution function (*g*(*r*), plotted against *r*/*σ* where *σ≠*2*R* is the mean interparticle distance) calculated from the tracked particle co-ordinates. The experimental *g*(*r*) value was compared with the theoretically calculated value for a 2D hexagonal lattice. d) Rotator phase as observed by an *xz* scan. e) Confocal image of the fcc rotator phase of hard PMMA particles in salt-saturated CHB. f) Confocal image of rotationally disordered glass. g) Scanning electron micrograph of a rotationally disordered fcc crystal on a substrate. Scale bars: 5 μm.

To shed light on how the range of the repulsive double-layer interactions influences the phase behavior, we decreased the electric double layer thickness around the particles by adding a salt, such as tetrabutylammonium bromide. To achieve hard particle interactions, we dispersed the particles in salt-saturated (260 μm) CHB. We estimated the Debye screening length (*κR*≈20, *R* is radius of the particle) corresponding to this salt concentration. As a result of hard particle interactions, the 3D rotator phase transformed into a defective non-rotator crystalline phase as shown in Figure 3 e. If the particles were not given enough time to rearrange themselves, then the particles completely lost their long-range positional order and the system became a rotationally disordered glass (Figure 3 f). We achieved this state by centrifugation of a dilute (*ϕ*≈0.08) sample for 40 minutes at 2000 rpm. When the particles were dried on a flat substrate, we observed orientationally disordered fcc crystals as shown in Figure 3 g. It is noteworthy that the particles were not arranged with their flat facets in a side-by-side fashion because the drying-induced forces were much stronger than thermal energy, and therefore, the particles did not have time to reorient their facets.

Finally, we show that facets can induce directionality in the interparticle interactions by means of depletion attractions by adding non-adsorbing polymer *(*1.0 wt % of polystyrene polymers; for details see the Supporting Information) to the system. It is clear from the possible increase in overlap volumes that the depletion force should be much more effective between flat interfaces than between curved interfaces.[3,12] As a consequence, aggregates composed of short linear segments and closed loops were observed at a low particle concentration (Figure 4 a) whereas the 3D gel network (Figure 4 b, c) was observed at a high particle concentration.

**Figure 4:**
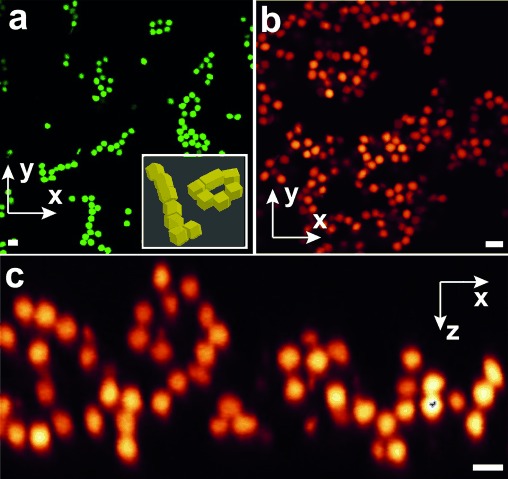
Confocal micrographs of clusters of particles formed by depletion attractions. a)  Particles aggregated in short linear segments and closed loops at low particle concentrations as observed in an *xy* confocal micrograph. Inset: conjectural schematic interpretation of internal structures of linear and closed loops. b, c) A network of particles at high particle concentrations is revealed by the *xy* and *xz* confocal images. Scale bars: 5 μm.

In conclusion, we have developed an effective, yet simple method for the fabrication of polyhedron-shaped polymeric particles that reflect the local Voronoi cell of the sphere packing that was originally adopted by the polymer spheres. Moreover, with this method, the particle shape could be tuned by varying the sintering time. We were able to make round particles with flat patches by stopping at the early stages of the heat-induced deformation process. This method is quite general as it relies only on an initial 3D assembled structure that consists of spherical particles; for such particles, several crystal structures have already been realized. With this bulk method, gram quantities of the particles can easily be obtained. As an illustration, we demonstrated the phase behavior of rhombic dodecahedron particles at different salt concentrations. We observed the 3D rotator phase at low salt concentrations, whereas the particles assembled into a non-rotator phase at high salt concentrations. We further demonstrated increased directional interactions by means of depletion attractions between the flat facets of the particles. Our method was demonstrated for PMMA particles; however, as it only makes use of the surface tension of the particles and as polymer exchange can be drastically reduced by crosslinking, it should also apply to other cross-linked polymer systems. Our procedure indeed also works with cross-linked polystyrene (PS) particles (Figure S5). Furthermore, the present paper focused on particles because we intend to use them in real-space confocal studies, but the method should work just as well with particles with sizes all over the colloidal domain as the surface tensions of even particles with a size of 10 nm are still much larger than *k*_B_*T*. Because of its simplicity, our thermal-annealing method opens the possibility for a wide range of amorphous polyhedral particle shapes. We believe that the characteristics of the dense packing arrangement and the larger interfaces between the facets make the polyhedral particles suitable building blocks for new materials. Both fundamental and applied studies on the utilization of these new polyhedral particles should thus yield interesting results.

## Experimental Section

PMMA particles were synthesized by dispersion polymerization, covalently labelled with the fluorescent dye 7-nitrobenzo-2-oxa-1,3-diazol (NBD) or rhodamine isothiocyanate (RITC), and sterically stabilized with poly(12-hydroxystearic acid), which was grafted onto the PMMA backbone forming a comb-graft steric stabilizer.[16] The PMMA spheres had a diameter of 2.6 μm. The particle size and polydispersity (4 %) were determined by static light scattering (SLS). The particles were dispersed in cyclohexyl bromide (CHB, Fluka) saturated with tetrabutylammonium bromide (TBAB, Sigma). In this dispersion, the particles were nearly refractive-index-matched, and they behaved like hard spheres.[19] We used rectangular capillaries of 0.1 mm×1.0 mm and 0.1 mm×2.0 mm (VitroCom, UK). After filling the cell with the colloidal suspension, we sealed both ends of the capillary with UV-curing optical adhesive (Norland No. 68) and cured the glue with UV light (*λ*=350 nm, UVGL-58 UV lamp). We studied the particle dynamics by means of confocal laser scanning microscopy (Leica TCS SP2). We estimated the Debye screening length of our suspensions by measuring the conductivity of the CHB (with a Radiometer analytical CDM 230 conductivity meter) and then applying Walden′s rule.[19] A sonicator of Branson, Model 8510 was used. We imaged the dried samples by a scanning electron microscopy (FEI Phenom scanning electron microscope). The particle morphology was investigated by atomic force microscopy (Dimension 3100, Bruker).
