# Supplementary material for: Fabrication of Polyhedral Particles from Spherical Colloids and Their Self-Assembly into Rotator Phases
Source: Angew Chem Int Ed Engl. 2014 Nov 3;53(50):13830–4. doi: 10.1002/anie.201409594 (PMC4502970; doi:10.1002/anie.201409594)
Supplement: Supplementary file 1 [file anie0053-13830-sd1.pdf]

Angewandte Chemie
Eine Zeitschrift der Gesellschaft Deutscher Chemiker

Supporting Information
© Wiley-VCH 2014
69451 Weinheim, Germany

Fabrication of Polyhedral Particles from Spherical Colloids and Their Self-Assembly into Rotator Phases**

Hanumantha Rao Vutukuri, Arnout Imhof, and Alfons van Blaaderen**

anie_201409594_sm_miscellaneous_information.pdf
anie_201409594_sm_movie.avi

Supplementary movie caption:

Movie S1: This movie shows Brown motion of rhombic dodecahedron particles in a FCC (110) rotator or plastic crystal phase. The movie was acquired at 1 frame per 3 second and it is played at 7 frames per second.

Atomic Force Microscopy measurements:

The morphology of patchy and polyhedron (Figures 2b&c) particles was investigated using atomic force microscopy (AFM, Dimension 3100, Bruker) in tapping mode. Samples for AFM were prepared by applying a drop of the particles in hexane onto a microscope slide. AFM images of typical patchy and polyhedron particles are shown in Figures S1a&d. These AFM measurements are indeed consistent with the SEM measurements. To measure the roughness on the flattened particle surfaces we first drew a line along a patch of the particle (Figure S1b), and also along the facets of a polyhedron (Figure S1e). We then measured the roughness by plotting the topographic profiles (SPIP6.3 software) along the blue lines shown in Figures S1c&f. The root mean-squared roughness was 9 nm for the patchy particles (Figure S1c), and 4 nm for polyhedral particles (Figure S1f). Although the roughness of the patch is a bit higher than the facets value, the patchy can also be considered as flat especially considering their micron-sizes.

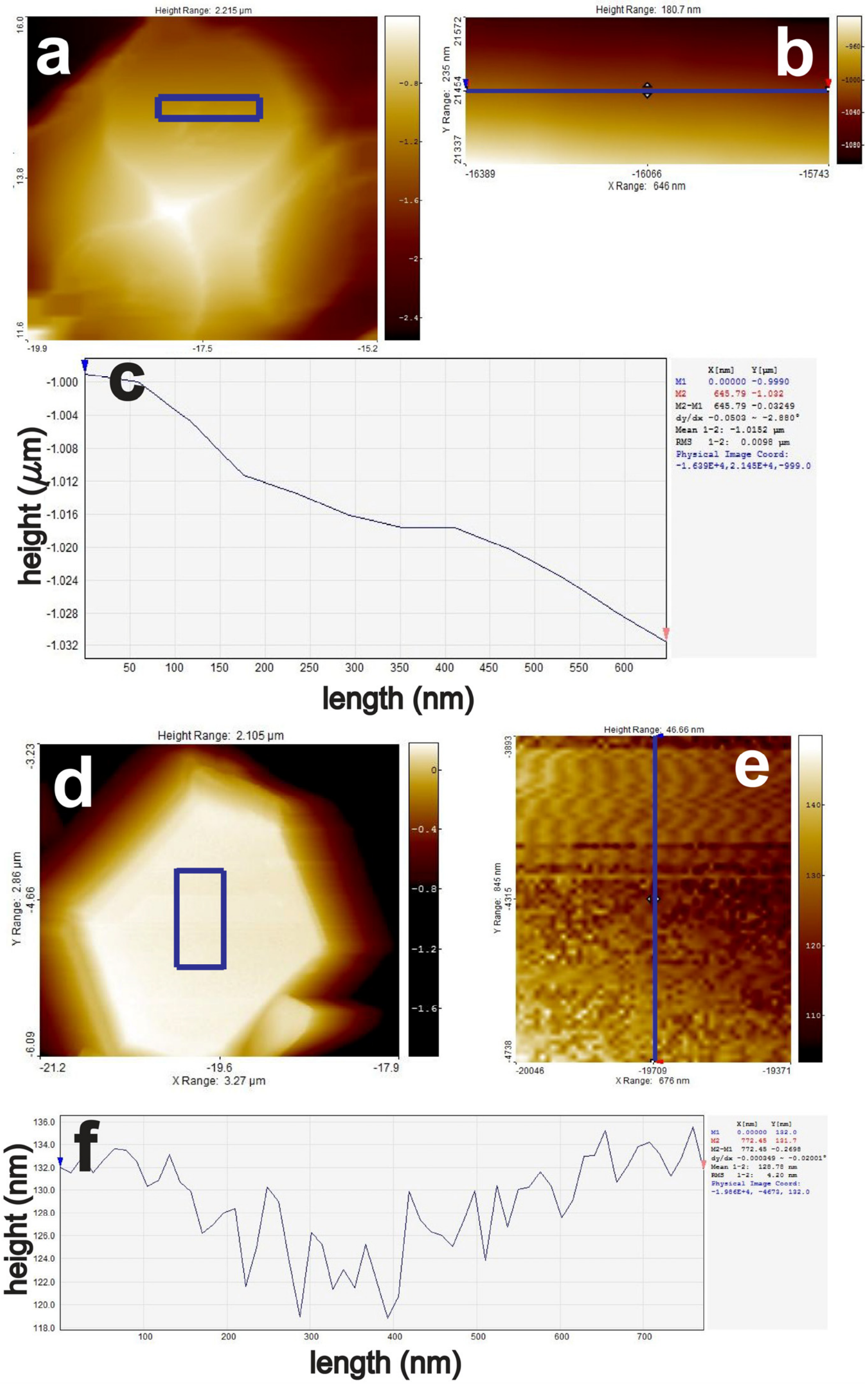

Figure S1. AFM images of typical patchy (a), and rhombic dodecahedron shaped (d) particles. (b&e), the magnified view of the figures a&d. Blue boxes represent where the roughness was measured. Images (c) and (f) show the height profiles taken along the lines drawn in the 2D images for patchy (b), and for facets (e), respectively.

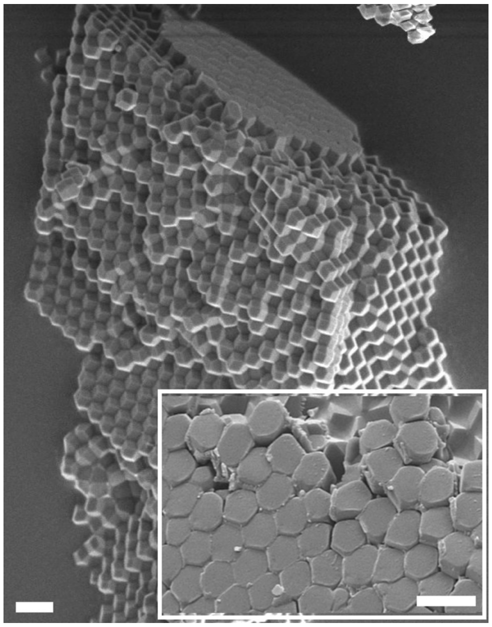

Figure S2: Scanning electron micrograph of dried polyhedral PMMA particles that were annealed for about 10mins in the presence of the solvent at 75 ºC. The lower inset illustrates that particles in direct contact with the wall become more flattened. Scale bar is 5.0 µm.

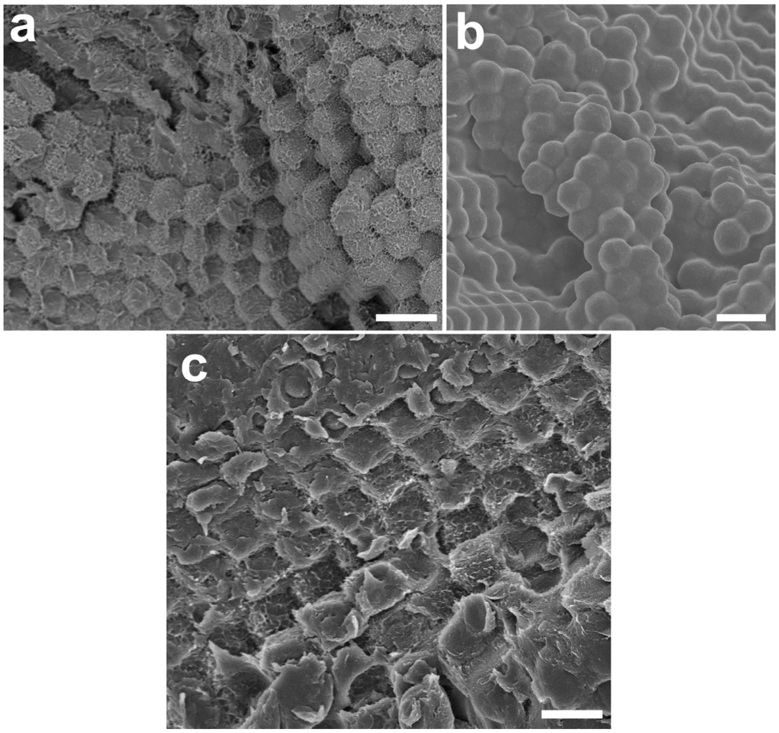

FigureS3. SEM images reveal the neck formation between the neighboring particles at the different stages of thermal sintering. (a) t_h = 2-3 mins, (b) t_h = 5-6 mins, and (c) t_h = 10-12 mins, respectively. The mass transport between the neighboring particles takes place when the

un-crosslinked particles are sterically stabilized by adsorbtion of the stabilizer and it is thus not chemically bonded with PMMA polymer chains forming the particle. Scale bar is 5 μm.

Fabrication of body centred tetragonal crystals (BCT):
Our colloidal suspension consisted of positively charged 2.6 *μm* diameter PMMA particles (dielectric constant, $\varepsilon_p \approx 2.6$) in a nearly refractive-index matching organic solvent cyclohexyl bromide ($\varepsilon_s \approx 7.9$). The particle volume fraction was $\varphi = 0.25$. The suspension was then introduced into a thin indium tin oxide coated electric cell[1,2] and subsequently exposed to a strong AC field ($\boldsymbol{E}_{rms} = 0.85\ V\,\mu m^{-1}$, $f = 1\ MHz$, where $\boldsymbol{E}_{rms}$ is the root-mean-square electric field strength, and f is the frequency). After 2-3 days, large single-domain bct crystallites that did not have a layer of colloidal fluid on top were observed. Next, the crystals were heat treated by immersing the electric cell into a hot water bath at 75 *ºC*, which is well below the glass transition temperature (T_g = 140-145 *ºC*) of PMMA for about 5 mins. We kept the field on for 20-30mins while the sample cell was allowed to cool down to room temperature. We note that the heat-treated assembled structures remained stable in the liquid even after the electric field was turned off. We carefully opened the cell and then dried the structure for about 2-3 days at room temperature.

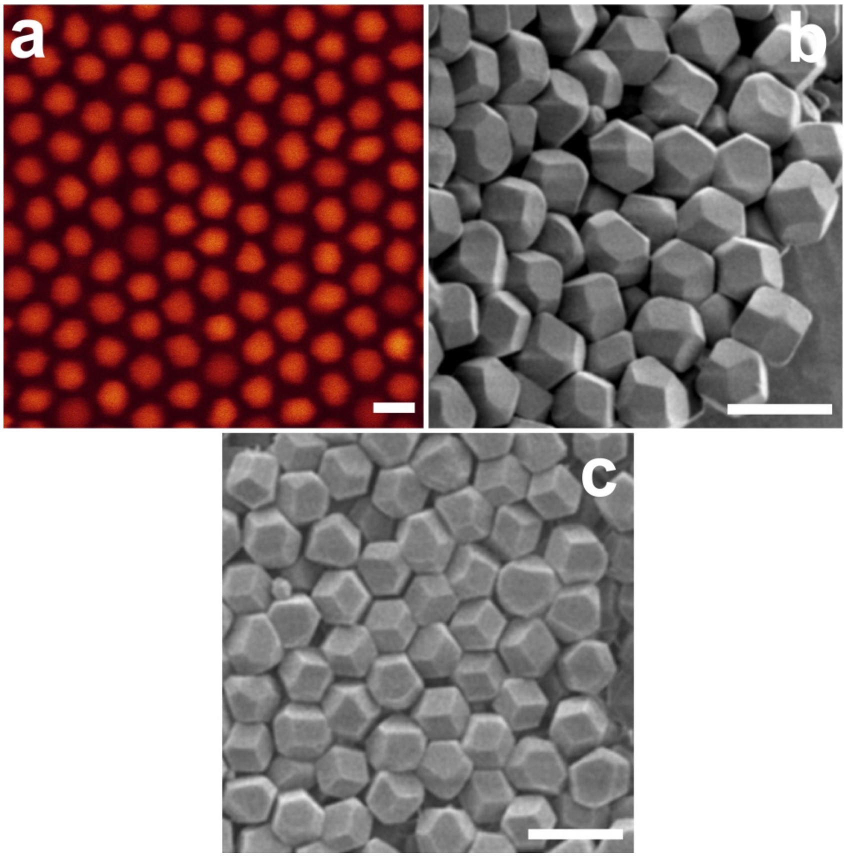

Figure S4. (a), Confocal micrograph of 3D rotator phase reveals the individual particles that are separated from the deformed supercrystal and redispersed completely unaggregated into the solvent. (b&c), SEM images of the dried and deformed particles that had been dispersed in CHB for about 2-3 weeks. There is no mass transport between the neighboring particles when the particles are sterically stabilized and chemically bonded with PMMA polymer chains. The

particles indeed also retained their deformed shape over time even in this solvent that slightly swells the particles.[2-3] Scale bar is 5.0 μm.

Depletion attractions:
We induced stronger directionality into the particle interactions by means of adding non-adsorbing polymer to the system. When the surfaces of two large particles come closer together than about the radius of gyration of the polymer, the polymer will be excluded from the region between the particles. Overlap of these excluded volumes induces an attractive force between the particles. It is clear from the basic geometry that the overlap volume between two flat interfaces is much larger and therefore the attractions are significantly more effective than between curved interfaces.[4-6] Polystyrene polymers (M_w = 350,000, Sigma Aldrich) were added to induce depletion attractions between the particles. 1.0 *wt*% of polystyrene was added to the system.

Polystyrene polyhedral particles fabrication:
We synthesized cross-linked and sterically stabilized polystyrene (PS) particles by dispersion polymerization.[7] We used cross-linked (3 %, w/w, divinylbenzene) and polyvinylpyrrolidone (PVP, M_w = 10 kg/mol, Sigma) stabilized 2.30 μm sized polystyrene (PS) particles in dimethyl sulfoxide (DMSO, Fluka). Several methods are available for growing colloidal crystals. We chose a simple method, crystallization induced by sedimentation. The suspension, consisting of 15% by volume of particles was transferred to a capillary cell with a 0.2 *mm X* 2.0 *mm* cross section and of a desired length (~ 10 *cm*) oriented in an upright position. After 1-2 weeks, a large random hexagonal closed packed crystal was observed. Next, we wet-sintered the self-assembled cross-linked PS particle for 6-8 mins at 65 ºC (T_g = 120 ºC). The resulted particle shape as shown in Figure S5.

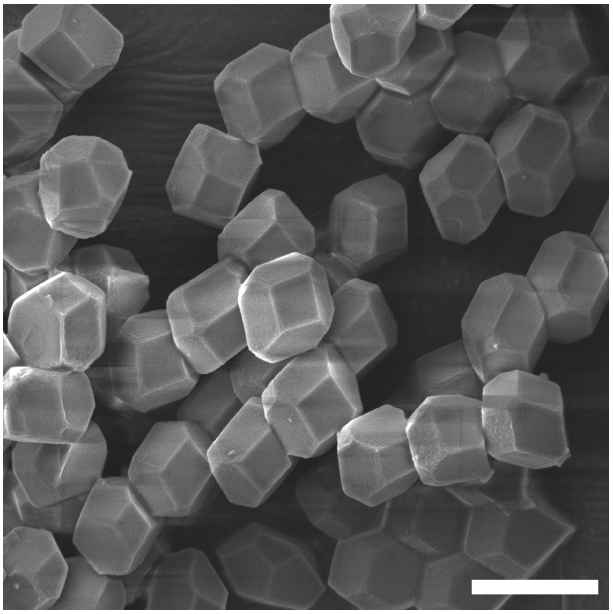

Figure S5. SEM micrograph of polyhedral cross-linked polystyrene particles. Scale bar is 5 μm.

References:

1. Yethiraj, A.; van Blaaderen, A. *Nature* **2003,** 421, 513-517.
2. Vutukuri, H. R.; Stiefelhagen, J.; Vissers, T.; Imhof, A.; van Blaaderen, A. *Adv. Mater.* **2012,** 24, 412-416.
3. Bosma, G.; Pathmamanoharan, C.; de Hoog, E. H. A.; Kegel, W. K.; van Blaaderen, A.; Lekkerkerker, H. N. W. *J. Colloid. Interf. Sci.* **2002,** 245, 292-300.
4. Badaire, S.; Cottin-Bizonne, C.; Woody, J. W.; Yang, A.; Stroock, A. D. *J. Am. Chem. Soc.* **2007,** 129, 40-41.
5. Sacanna, S.; Irvine, W. T. M.; Chaikin, P. M.; Pine, D. J. *Nature* **2010,** 464, 575-578.
6. Kraft, D. J.; Ni, R.; Smallenburg, F.; Hermes, M.; Yoon, K.; Weitz, D. A.; van Blaaderen, A.; Groenewold, J.; Dijkstra, M.; Kegel, W. K. *Proc. Nat. Acad. Sci. USA* **2012,** 109, 10787-10792.
7. Song, J. –S.; Tronc, F.; Winnik, M. A. *J. Am. Chem. Soc.* **2004**, *126*, 6562-6563.
